# Dexamethasone Inhibits *S. aureus*-Induced Neutrophil Extracellular Pathogen-Killing Mechanism, Possibly through Toll-Like Receptor Regulation

**DOI:** 10.3389/fimmu.2017.00060

**Published:** 2017-02-09

**Authors:** Ting Wan, Yingying Zhao, Fangli Fan, Renjian Hu, Xiuming Jin

**Affiliations:** ^1^Eye Center, Second Affiliated Hospital, School of Medicine, Zhejiang University, Hangzhou, China

**Keywords:** neutrophil extracellular traps, dexamethasone, TLRs, *S. aureus*, PMA

## Abstract

Neutrophils release neutrophil extracellular traps (NETs) in a pathogen-killing process called NETosis. Excessive NETs formation, however, is implicated in disease pathogenesis. Therefore, to understand how NETosis is regulated, we examined the effect of dexamethasone (DXM), an anti-inflammatory drug, on this process and the role of toll-like receptors (TLRs). We stimulated human neutrophils with phorbol 12-myristate 13-acetate (PMA) or *Staphylococcus aureus* (*S. aureus*) and quantified NETs formation. We also examined the effect of DXM on the bactericidal effect of NETs and the role of reactive oxygen species (ROS) and nuclear factor (NF)-κB in DXM-regulated NETosis. DXM significantly inhibited *S. aureus*-induced NETosis and extracellular bacterial killing. ROS production and NF-κB activation were not involved in DXM-regulated NETosis. TLR2 and TLR4, but not TLR5 or TLR6, modified *S. aureus*-induced NETs formation. Neither DXM nor TLRs were involved in PMA-induced NETosis. Furthermore, TLR2 and TLR4 agonists rescued DXM-inhibited NETosis, and neither TLR2 nor TLR4 antagonists could further inhibit NETosis reduction induced by DXM, indicating that DXM may inhibit NETosis by regulating TLR2 and TLR4. In conclusion, the mechanisms of *S. aureus*- and PMA-induced NETosis are different. DXM decreases NETs formation independently of oxidant production and NF-κB phosphorylation and possibly *via* a TLR-dependent mechanism.

## Introduction

Neutrophils are the most abundant leukocytes in human blood and play an essential role in innate immunity since they are the first cells recruited to sites of infection and inflammation (1). They engulf microorganisms or opsonized particles and degrade them intracellularly as well as releasing microbicidal proteins and reactive oxygen species (ROS) (2). Recently, these cells have been shown to release structures called neutrophil extracellular traps (NETs), which consist of chromatin along with histones and many granular antimicrobial proteins—including elastase, myeloperoxidase, and calprotectin; this is a novel extracellular pathogen-killing mechanism described as NETosis (3–5).

Although NETosis contributes to pathogen control, it is essential for the balance between the formation and removal of NETs to be regulated to ensure tissue homeostasis, because large amounts of NETs may contribute to collateral damage within inflamed tissues. Excessive amounts of NETs are associated with the pathogenesis of inflammatory and autoimmune diseases, including preeclampsia (6), cystic fibrosis (7), and lupus (8). Moreover, NETs have been observed to act as a scaffold for thrombus formation (9, 10), which is increasingly being recognized as a critical phenomenon linking inflammation with venous thrombosis. Therefore, NETosis is a double-edged sword: while it is an effective first-line antimicrobial mechanism, it might also lead to organ failure and death if it is unregulated. Hence, it is important to understand the mechanism of NETs regulation, but little information is available about this topic thus far.

Since an inflammatory microenvironment is essential for NETs formation, we believed that using glucocorticoids, which are potent anti-inflammatory drugs, can help elucidate how NETs formation is regulated. They are commonly used to resolve inflammation and are closely related to neutrophil function. They have been shown to inhibit neutrophil apoptosis and cytokine release during inflammation (11) and are also associated with many neutrophil functions, including chemotaxis, migration, and phagocytosis (12). Therefore, we examined the effect of a commonly used glucocorticoid drug, dexamethasone (DXM), on NETs formation. On the other hand, toll-like receptors (TLRs), which are essential pattern-recognizing receptors (PRRs) that mediate the recognition of microbial structures, have been reported to activate neutrophil extracellular traps to ensnare bacteria in septic blood (13). Moreover, most of the TLRs were reported to be expressed in neutrophils and were involved in neutrophils activation (14). So, we also investigated the role of different TLRs in NETs formation.

We found that DXM significantly inhibited NETs formation induced by *Staphylococcus aureus* (*S. aureus*) but not that induced by phorbol 12-myristate 13-acetate (PMA), which suggested that DXM can serve as a potential drug to regulate NETosis. In addition, the modulation of TLR-2 and TLR4 had an effect on NETs production, thus indicating the involvement of TLRs in this process.

## Materials and Methods

### Reagents

Phorbol 12-myristate 13-acetate, DXM, DNase I, cytochalasin D, and dichlorofluorescein diacetate (DCF-DA) were purchased from Sigma-Aldrich (St. Louis, MO, USA); Percoll, from GE Healthcare (Little Chalfont, UK); and TLR agonists and TLR antagonists, from InvivoGen (San Diego, CA, USA). Anti-histone H2B and neutrophil elastase antibodies, anti-glyceraldehyde-3-phosphate dehydrogenase (GAPDH) antibody, anti-phosphorylated nuclear factor κB (anti-p-NF-κB, p65) antibody, secondary antibodies coupled to AF488 or AF555, and horseradish peroxidase (HRP) secondary antibody were purchased from Santa Cruz Biotechnology (CA, USA). SYTOX Green, Luria broth, Quant-iT PicoGreen double-stranded deoxyribonucleic acid (dsDNA) assay kit and micro-plates were purchased from ThermoFisher Scientific (Basingstoke, UK).

### Isolation of Human Neutrophils

Neutrophils were isolated from the peripheral blood of fasting healthy donors by Percoll gradient centrifugation, as previously described (7). For those donors, comprehensive history and physical examination were performed, basic laboratory tests were used to exclude occult disease. This study was conducted according to the principles expressed in the Declaration of Helsinki. Ethical approval was obtained from the Ethics Committee of Affiliated Second Hospital, School of Medicine, Zhejiang University, China. All participants provided written informed consent for the collection of samples and subsequent analyses. For each donor, 10–30 ml blood was drawn according to the need of different assays. Bloods from at least three donors were used to repeat the same assay. Cell suspensions contained >96% neutrophils, as determined by Wright–Giemsa staining, with 98% cell viability as determined by Trypan blue staining. The cells (4 × 10^5^/ml) were re-suspended in RPMI 1640 medium supplemented with bovine serum albumin (2%).

### Neutrophils Stimulation

Neutrophils (2 × 10^5^ cells/well in 500 µl) were stimulated with PMA (50 nM) or *S. aureus* (multiplicity of infection = 10) and placed in a humidified incubator at 37°C with CO_2_ (5%) for 120 min. In some experiments, neutrophils were first incubated for 120 min with DXM (10 µM), TLR2 agonist (HKLM, 10^8^ CFU/ml), TLR4 agonist (LPS, 1 µg/ml), TLR5 agonist (FSL-ST, 1 µg/ml), TLR6 agonist (FSL-1, 1 µg/ml), TLR neutralizing antibodies as antagonists (TLR2, 4, 5, 6 antibody, 1 µg/ml), or vehicle (controls). Stock solutions of DXM, TLR agonists, and TLR antagonists were prepared in DMSO and were further diluted in RPMI 1640 medium. The final DMSO concentration (0.1% v/v) did not have a toxic effect. All drugs were freshly prepared for each experiment.

### NETs Formation Assay

After stimulation, cells were fixed with 4% PFA, blocked with 3% normal donkey serum and 0.05% Tween 20 in phosphate-buffered saline (PBS), and incubated with the primary antibodies anti-H2B and anti-neutrophil elastase, which were detected with secondary antibodies coupled to AF488 or AF555. Isotype-matched controls were used. For DNA detection, 4′, 6′-diamidino-2-phenylindole (DAPI) was used. Specimens were mounted and analyzed under a confocal microscope (Olympus IX-50).

Neutrophil extracellular traps were also examined using the membrane-impermeable DNA-binding dye SYTOX green (Molecular Probes, Invitrogen Life Technologies). SYTOX green (5 µM) was added to the cultures after specific periods of incubation, and the cultures observed 5 min later. In one case, DNase I (100 U/ml) was added for 10 min to degrade the NETs structure as control. To visualize NETs, live-cell cultures were imaged with an inverted fluorescence microscope (Olympus IX-50).

### Bacterial Culture

*Staphylococcus aureus* (ATCC 25923) was cultured overnight in Luria–Bertani (LB) broth (37°C, 200 rpm), harvested by centrifugation, washed, and suspended in PBS. Bacterial growth was quantified at A600 and the cell number determined using a standard curve based on colony counts. Stationary-phase bacteria were used for all experiments.

### Quantification of Extracellular DNA

The levels of extracellular DNA in supernatants were quantified using Quant-iT PicoGreen dsDNA assay kit according to the manufacturer’s instructions. PicoGreen is a cell-impermeable dye that binds to extracellular dsDNA without staining live cells. Fluorescence intensity was measured on a SpectraMax M3 (Molecular Devices) fluorescent plate reader at an excitation wavelength of 480 nm and an emission wavelength of 520 nm, with a 515-nm emission cutoff filter. The calibration curve was constructed using a standard dsDNA of a known concentration.

### Bacterial Survival Assay of NETs

A bacterial survival assay was performed as described in earlier studies (15). Neutrophils (1 × 10^6^ cells/well in 200 µl) were pre-incubated with or without DXM for 2 h and then treated with 50 nM PMA or left untreated for another 2 h at 37°C and 5% CO_2_. NETs killing was examined by inhibiting phagocytic killing by the addition of 100 µg/ml cytochalasin D for 15 min before the addition of bacteria. After 1 h at 37°C, neutrophils and clumped NETs were disrupted by the addition of 0.01% Triton X-100 and three passes through a 25-gauge needle. Following serial dilution, bacteria were plated on LB plates for colony counting. After overnight incubation at 37°C, the number of colony-forming units (CFU) was determined. Zero killing was defined by control samples consisting of RPMI 1640. Killing efficacy was determined by subtracting the CFU of indicated treatment from control group.

### ROS Production

Neutrophils were incubated in PBS (Ca^2+^- and Mg^2+^-free) with 10 µM DCF-DA (Sigma) at 37°C for 20 min. Subsequently, they were pelleted, washed in PBS three times, and transferred to a 96-well plate (1 × 10^6^ cells/well in 100 µl). They were then stimulated with *S. aureus* for 1 h (some cells were pretreated with DXM for 120 min), and fluorescence was measured using SpectraMax M3 fluorescent plate reader at an excitation wavelength of 480 nm and an emission wavelength of 520 nm.

### Immunoblotting

The neutrophils (3 × 10^6^ cells/well in 500 µl) were pre-incubated with or without DXM for 2 h and then stimulated for another 2 h with *S. aureus*. Cell lysates were prepared using 1× loading buffer and boiled. Samples were then frozen at −80°C until use. Equal amounts of proteins were run on 12% sodium dodecyl sulphate-polyacrylamide gel and then electrotransferred onto polyvinylidenefluoride membranes. After blocking with 5% bovine serum albumin, membranes were incubated with phospho-NF-κBp65 and anti-GAPDH antibody overnight at 4°C, and then with HRP-conjugated secondary antibody for 2 h at room temperature. Protein bands were visualized by enhanced chemiluminescence. The gray degree of protein bands was detected by image J, and the value of p-NF-κB p65/GAPDH was calculated.

### Statistical Analysis

Statistical analyses were performed using GraphPad Prism 6.1. Data are expressed as mean ± SE of individual samples. For two-group comparison, Student’s *t*-test was applied for normally distributed data. The comparisons between multiple groups were performed using one-way ANOVA, followed by a Bonferroni’s post-test. The significance threshold was set at 0.05.

## Results

### NETs Formation in Response to PMA and Bacterial Stimulation

**Figure 1:**
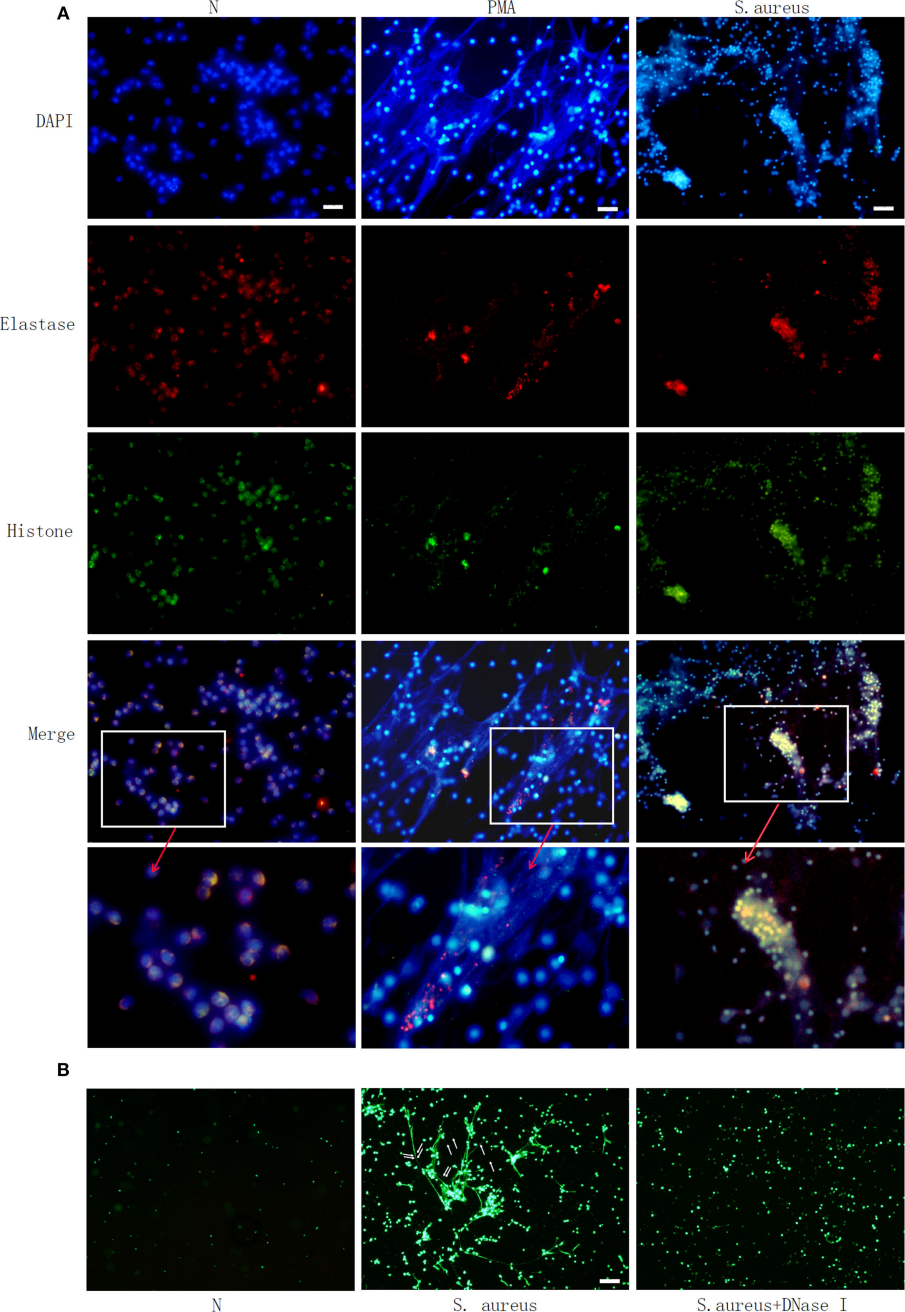
**Phorbol 12-myristate 13-acetate (PMA) and *Staphylococcus aureus* (*S. aureus*) stimulate neutrophil extracellular traps (NETs) formation in human neutrophil**. Human neutrophil suspended in media were treated with PMA (50 nM) or *S. aureus* at MOI of 10. Human neutrophil without treatment (N) was used as control. NETs formation was measured at 2 h. **(A)** Neutrophils were labelled with 4′, 6′-diamidino-2-phenylindole (DAPI) to identify DNA (blue) and with antibodies to identify neutrophil histone (green) and elastase (red). PMA and *S. aureus*-induced NETs formation. **(B)** *S. aureus* (indicated with arrow) were trapped in NETs and released when NETs structure was degraded by DNase I, as observed by SYTOX green staining Bar: 50 μm.

Microscopic observation clearly showed NETs structure, including neutrophil-derived proteins. Neutrophils were labeled with DAPI to identify DNA (blue) and with antibodies to identify neutrophil histone (green) and elastase (red) (Figure 1A). This confirmed PMA- or *S. aureus*-triggered NETs formation. SYTOX green staining further showed that bacteria were trapped in the web like structure and could be released when this NETs formation was degraded by Dnase I (Figure 1B).

### DXM Inhibits NETs Formation Induced by *S. aureus* But Not That Induced by PMA

**Figure 2:**
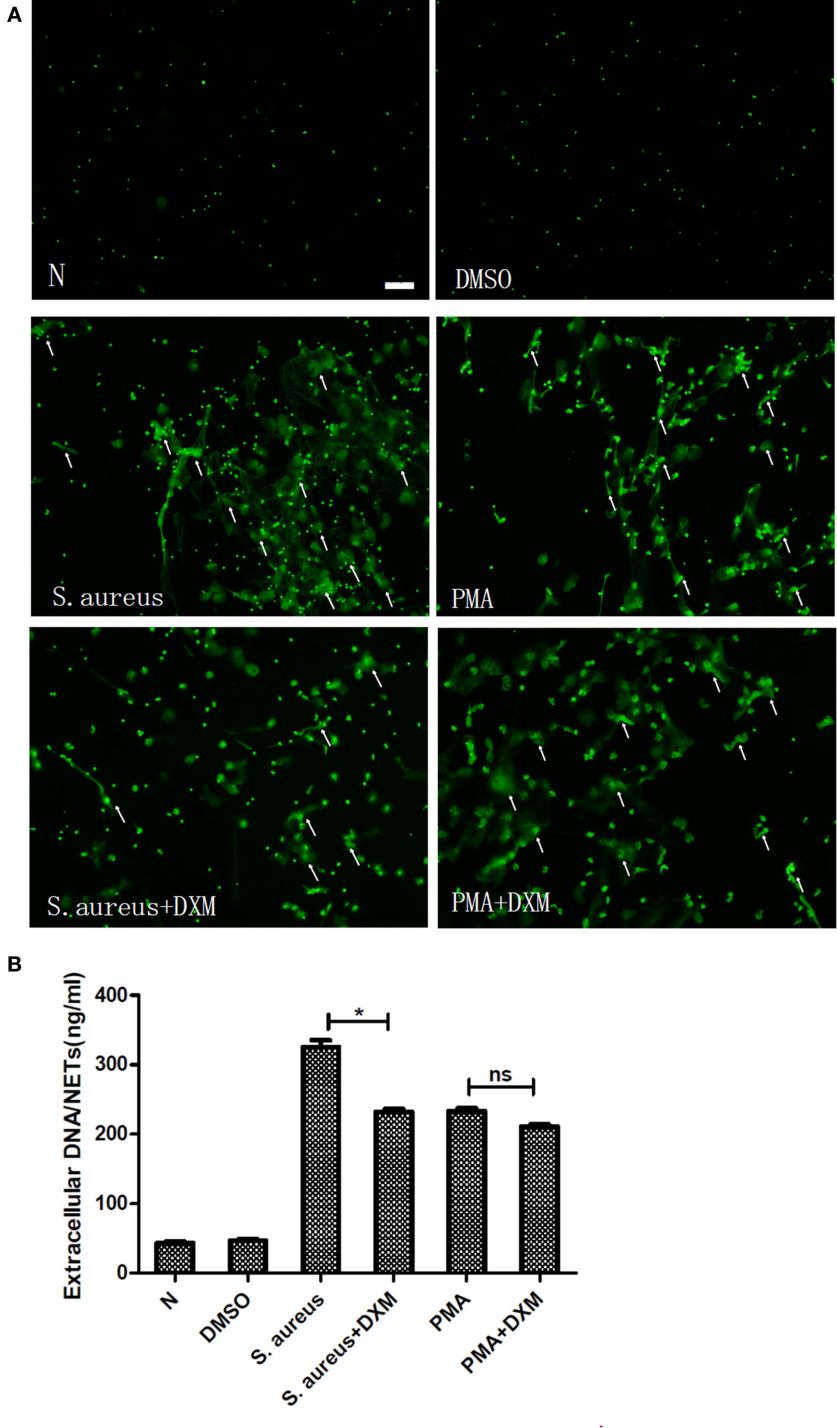
**Dexamethasone (DXM) inhibited neutrophil extracellular traps (NETs) formation induced by *Staphylococcus aureus* (*S. aureus*) but not that induced by phorbol 12-myristate 13-acetate (PMA)**. Human neutrophils suspended in media were pretreated with or without DXM (10 µM) for 2 h, and then NETs formation 2 h after stimulation with PMA or *S. aureus* was examined using the membrane-impermeable DNA-binding dye SYTOX green and quantified by Quant-iTPicoGreen double-stranded deoxyribonucleic acid assay kit. Neutrophils without any stimulation or treated with DMSO (0.1% v/v) were used as control. **(A)** DMSO (0.1% v/v) did not affect NETs formation. While dexamethasone did not modify NETs formation induced by PMA, it markedly inhibited that induced by *S. aureus*. Several typical NETs were indicated with arrows. Bar: 50 μm. **(B)** Quantification of extracellular DNA confirmed that it inhibited NETs formation induced by *S. aureus* but not that induced by PMA. Neutrophils (1 × 10^5^cells/well in 100 µl) were stimulated to form NETs, and the mean value of NETs amount in five replicated wells was adopted. The assay was repeated for three times with bloods from three different donors; error bars represent SEM. *p < 0.05 by ANOVA with Bonferroni’s post-test.

Fluorescence microscopy showed that DXM did not have any effect on the NETs formation induced by PMA but markedly inhibited that induced by *S. aureus* (Figure 2A). To further corroborate these, NETs formation was measured by quantifying the extracellular DNA in the supernatants. This experiment confirmed that *S. aureus*-induced formation of extracellular traps was significantly decreased by DXM (*p* < 0.05). In contrast, the amount of NETs formed after PMA induction was similar in controls and in DXM-treated neutrophils (*p* > 0.05) (Figure 2B). In addition, DMSO (0.1% v/v) in the solution of stimulates had no effect on NETs formation.

### DXM Decreases the Bactericidal Efficacy of NETs

**Figure 3:**
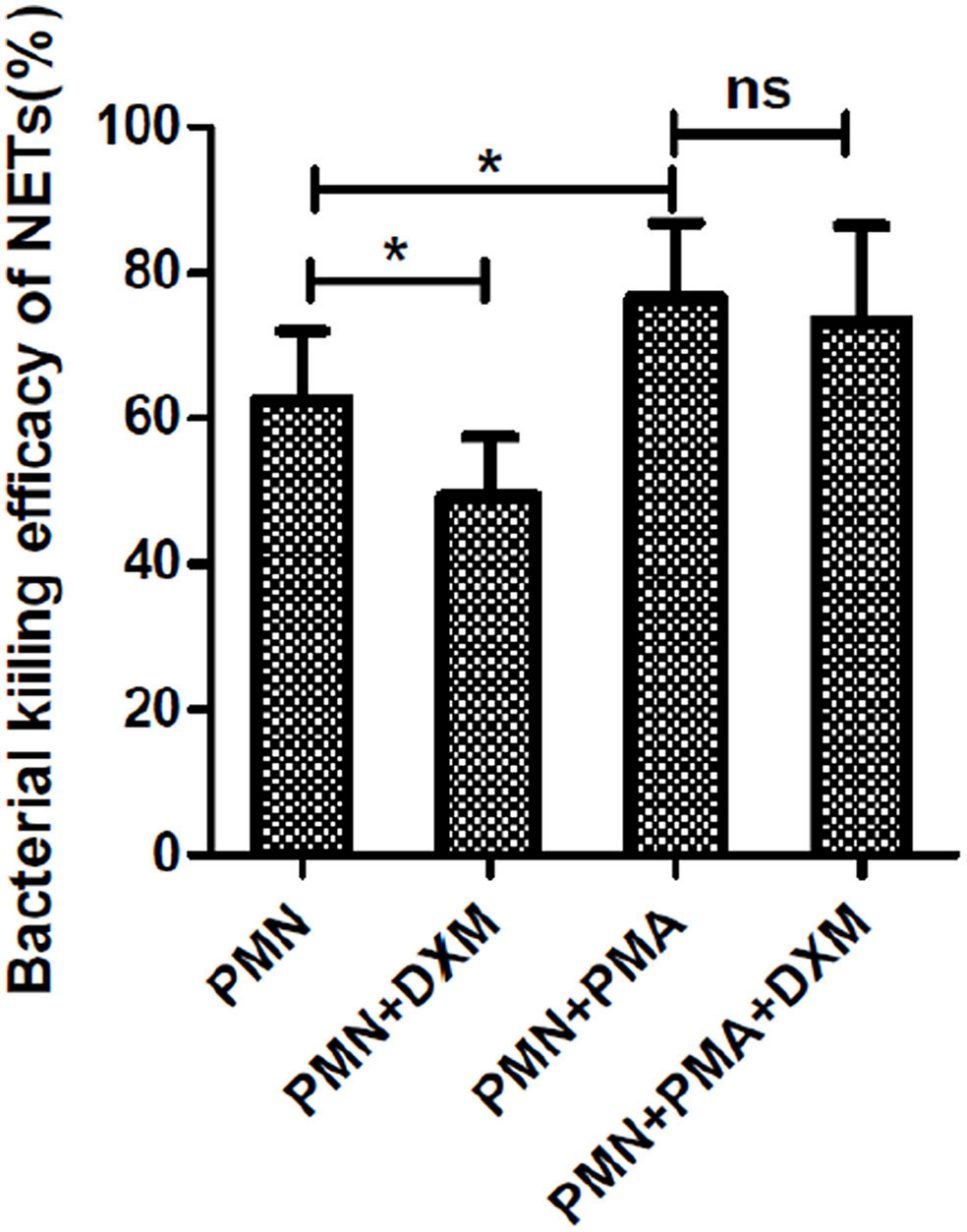
**Dexamethasone (DXM) inhibited the bactericidal efficacy of neutrophil extracellular traps (NETs)**. Neutrophils were pre-incubated with or without DXM for 2 h and then treated with 50 nM phorbol 12-myristate 13-acetate (PMA) or left untreated for another 2 h. One hour after addition of bacteria, colony-forming units (CFU) were determined by overnight incubation at 37°C following serial dilution. Zero killing was defined by control samples consisting of only media. Killing efficacy was determined by subtracting the CFU of indicated treatment from control groups. By using cytochalasin D to abrogate phagocytic killing, dexamethasone was found to significantly inhibit the bactericidal efficacy of NETs. However, dexamethasone could not inhibit PMA-activated bactericidal efficacy of NETs. The assay was repeated for nine times, each case in three wells; error bars represent SEM. *p < 0.05 by ANOVA with Bonferroni’s post-test.

Dexamethasone significantly decreased the bactericidal efficacy of NETs, following abrogating phagocytic killing by the addition of cytochalasin D (*p* < 0.05; Figure 3). However, if neutrophils were activated to form NETs by PMA, DXM treatment had no effect on the killing efficacy of NETs (*p* > 0.05).

### ROS and NF-κB Activation Are Not Involved in DXM-Regulated NETosis

**Figure 4:**
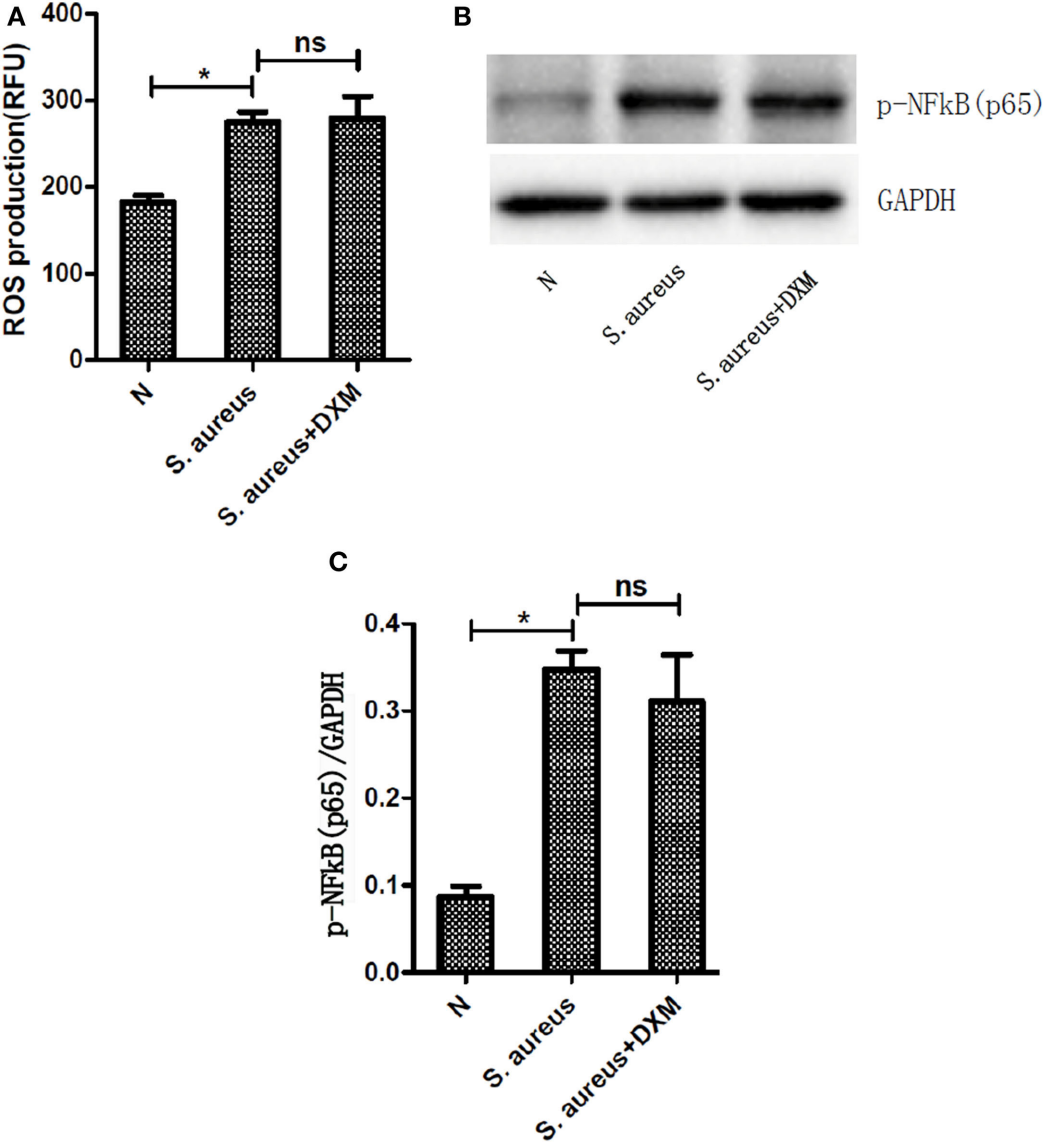
**Activation of reactive oxygen species (ROS) or nuclear factor (NF)-κB was not involved in dexamethasone-regulated NETosis**. Neutrophils were pretreated with or without dexamethasone (DXM) for 2 h and then stimulated with *Staphylococcus aureus* (*S. aureus*) for 1 h. ROS production were determined by dichlorofluorescein diacetate fluorescence and NF-κB activation were determined by Western blot. **(A)** *S. aureus* infection elicited significant neutrophil oxidative burst, but DXM treatment neither increased nor decreased this response. Data represent mean ± SEM of triplicate experiments. **(B)** NF-κB was activated when stimulated with *S. aureus* but not modified by dexamethasone. **(C)** Quantification showed that p-NF-κB (p65) expression was significantly higher when the cells were stimulated with *S. aureus*, but this effect was not modified by dexamethasone. Data represent mean ± SEM of triplicate experiments, *p < 0.05 by Student’s *t*-test.

Reactive oxygen species generation was first evaluated in resting neutrophils by performing a DCF-DA fluorescence assay. DCF-DA is a non-fluorescent molecule that becomes fluorescent in the presence of a wide variety of ROS, including superoxide anion and hydroxyl radicals (16). NETs formation has previously been reported to be dependent on or independent of ROS (17). In order to examine if DXM-regulated NETs formation is ROS-dependent, ROS production by activated neutrophils with or without DXM stimulation was measured. *S. aureus* infection elicited significant neutrophil oxidative burst, but DXM treatment neither increased nor decreased this response noticeably (Figure 4A).

The transcription factor NF-κB is a key regulator of inflammation and therefore plays a pivotal role in a wide range of inflammatory diseases (18). The phosphorylation of NF-κB has been believed to be involved in NETs generation (19). Therefore, we explored the role of DXM in the activation of NF-κB induced by *S. aureus*. The expression of p-NF-κB (p65) was significantly higher when the cells were stimulated with *S. aureus*, but this effect was not modified by DXM (Figures 4B,C).

### TLRs Are Involved in NETs Formation Induced by *S. aureus* But Not That Induced by PMA

**Figure 5:**
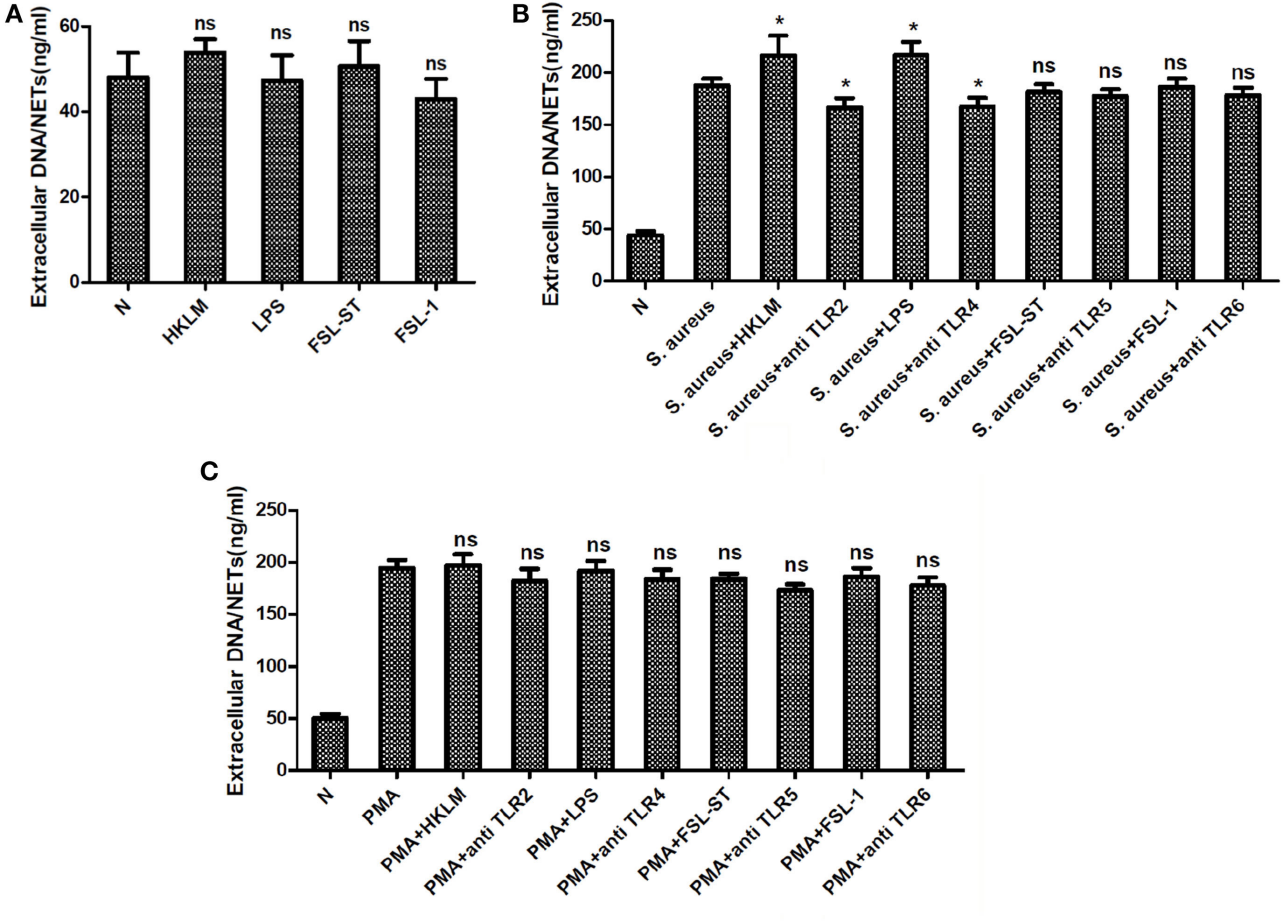
**Toll-like receptors (TLRs) were involved in *Staphylococcus aureus* (*S. aureus*)-induced but not phorbol 12-myristate 13-acetate (PMA)-induced neutrophil extracellular traps (NETs) formation**. Neutrophils were pretreated with TLRs agonist or antagonist, followed by PMA or *S. aureus* stimulation. NETs formation was quantified by Quant-iT PicoGreen double-stranded deoxyribonucleic acid assay kit. **(A)** None of TLR2 agonist (HKLM), TLR4 agonist (LPS), TLR5 agonist (FSL-ST), and TLR6 (FSL-1) agonist could induce NETs formation. **(B)** Treatment with TLR2 agonist (HKLM) and TLR4 agonists (LPS) significantly enhanced NETosis, and blocking TLR2 and TLR4 with neutralizing antibodies significantly reduced *S. aureus*-induced NETs formation. None of TLR5 agonist (FSL-ST), TLR6 agonist (FSL-1), and TLR5 and TLR6 neutralizing antibodies was involved in *S. aureus*-induced NETs formation. **(C)** TLRs were not involved in PMA-induced NETs formation. The assay was repeated for three times, each case in five wells, error bars represent SEM. Compared to *S. aureus* or PMA stimulation, *p < 0.05, ns = *p* > 0.05 by Student’s *t*-test.

Toll-like receptors are key PRRs, which are important in innate immune responses. Thus, we explored the role of TLRs in the formation of NETs. None of TLR2 agonist, TLR4 agonist, TLR5 agonist, and TLR6 agonist could induce NETs formation. However, TLR2 and TLR4 agonists significantly enhanced NETs formation induced by *S. aureus* but not that induced by PMA, as shown by the quantification of extracellular DNA. Moreover, blocking TLR2 and TLR4 with neutralizing antibodies significantly reduced the NETs formation induced by *S. aureus* but not that induced by PMA, as shown by quantification of extracellular DNA (Figure 5). Furthermore, neither the TLR5/TLR6 agonist nor the antagonist could modulate the formation of NETs.

### DXM May Modulate *S. aureus*-Induced NETs Formation through TLR2 and TLR4

**Figure 6:**
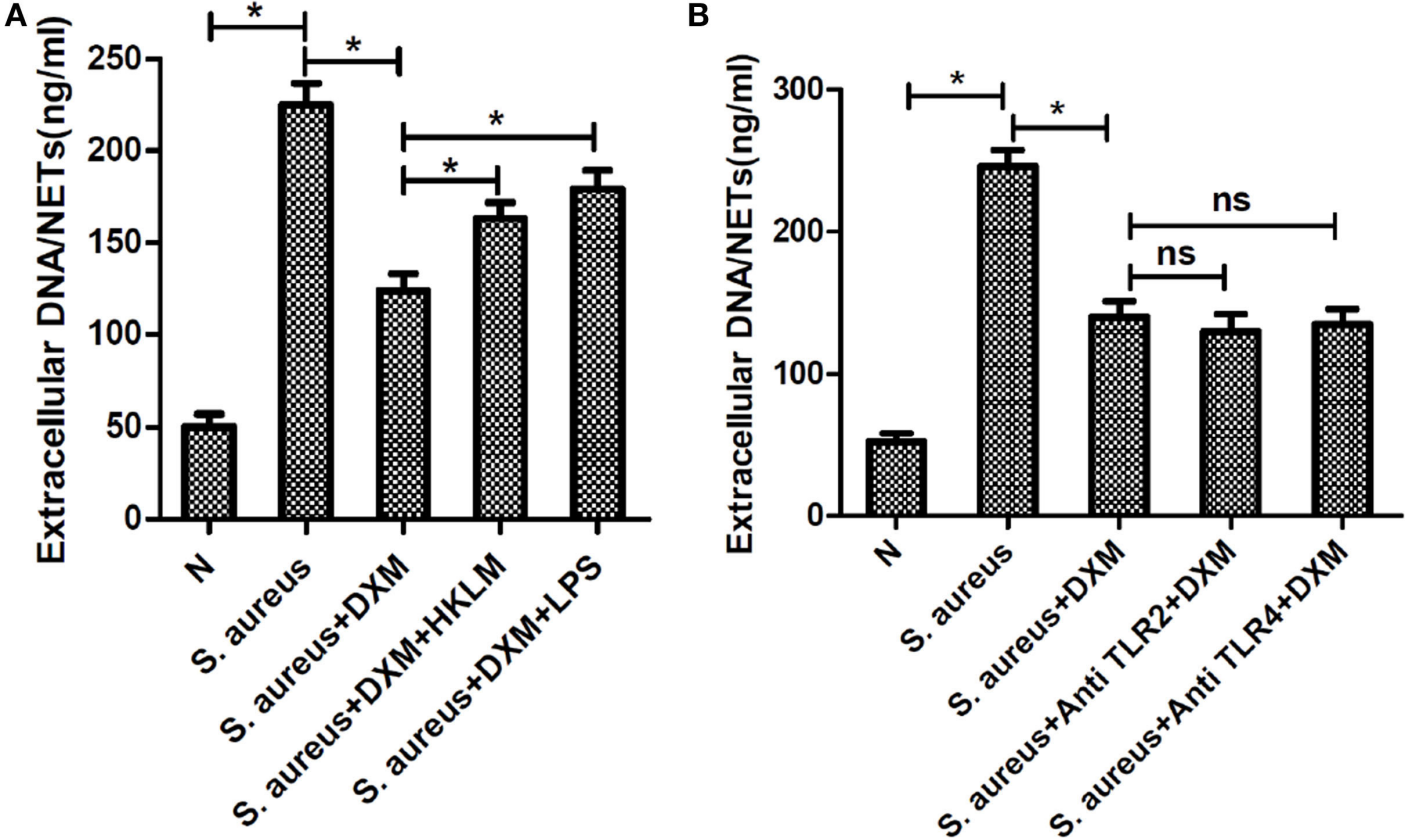
**Dexamethasone (DXM) may modulate *Staphylococcus aureus* (*S. aureus*)-induced neutrophil extracellular traps (NETs) formation through toll-like receptor (TLR)2 and TLR4**. **(A)** Neutrophils were pretreated with HKLM (TLR2 agonist) and LPS (TLR4 agonist), followed by DXM treatment and *S. aureus* stimulation. NETs formation was quantified by Quant-iTPicoGreen double-stranded deoxyribonucleic acid (dsDNA) assay kit. Both HKLM and LPS rescued dexamethasone-reduced NETs formation. **(B)** Neutrophils were pretreated with TLR2 and TLR4 antagonist, followed by DXM treatment and *S. aureus* stimulation. NETs formation was quantified by Quant-iTPicoGreen dsDNA assay kit. Neither TLR2 nor TLR4 antagonist could further decrease DXM induced NETosis reduction. The assay was repeated for three times, each case in five wells, error bars represent SEM. *p < 0.05 by Student’s *t*-test.

To explore the mechanism of DXM-modulated NETs formation, we first pre-incubated the cells with TLR agonists to examine the effect of TLRs on DXM-inhibited NETs formation. As expected, both HKLM (TLR2 agonist) and LPS (TLR4 agonist) rescued DXM-reduced NETs formation (Figure 6A). Moreover, neither TLR2 nor TLR4 antagonist could further decrease DXM-induced NETosis reduction (Figure 6B). While these findings suggested that DXM may modulate *S. aureus*-induced NETs formation through TLR2 and TLR4, further research is required to understand the precise mechanism.

## Discussion

NETosis, a recently identified mechanism of pathogen killing, helps in isolating and preventing the spread of invading bacteria, but the persistent formation or insufficient degradation of NETs can also cause injury to the host (8, 20). Since regulation of NETs formation is essential for tissue homeostasis, we aimed to determine the mechanisms and molecules underlying the regulation of this process.

A variety of stimuli promote NETs formation. In our study, NETs formation could be induced in neutrophils by both pharmacologic (PMA) and pathogenic (bacterial) stimuli, a finding that is in agreement with those of previous studies (21, 22). Although several signaling mechanisms responsible for NETs formation have been reported, critical regulatory elements remain unidentified. Since the findings from different studies often vary, it is possible that more than one mechanism exists. In this study, we observed that DXM-inhibited NETs formation induced by bacteria but not that induced by PMA. In addition, it markedly decreased the bactericidal ability of NETs. Thus far, DXM has not been reported to affect NETs formation induced by *S. aureus*. Lapponi reported that treatment of neutrophils with DXM had no effect on NETs formation induced by PMA or TNF-α (19). This is consistent with our observation that DXM was not required for the regulation of PMA-induced NETs formation. Other studies have suggested that NETs formation induced by different stimuli have distinct mechanisms. For example, Riyapa et al. (23) reported that when compared to the neutrophils of diabetic patients, those of normal individuals produced less PMA-induced NETs but the same amount of *S. aureus*-induced NETs. Parker et al. (24) hypothesized that whether NADPH oxidase and myeloperoxidase are required in NETs formation depends on the stimulus. These results prompted us to investigate whether different stimuli indeed have different underlying mechanisms. Our findings strongly suggested that bacteria and PMA regulate NETs formation through different pathways and that DXM may have an effect on NETs formation induced by bacteria but not on that induced by PMA.

Neutrophil extracellular traps formation has been shown to require NADPH oxidase activity as well as NF-κB activation. Our results verified the involvement of NADPH oxidase activity and NF-κB activation in the process of NETs formation. However, in contrast to our expectation, no change in ROS or pNF-κB levels was observed in DXM-treated neutrophils stimulated by *S. aureus*, which indicated that ROS and NF-κB signaling pathways were not involved in DXM-regulated NETs formation. NETosis was previously reported to be of two types: ROS dependent and ROS independent. Our study shows that DXM may modulate ROS-independent NETosis. Interestingly, DXM has been reported to inhibit calcium mobilization, which was shown to increase in LPS-treated cells (25). Therefore, DXM may regulate NETosis by modulating calcium mobilization, which is ROS independent. Moreover, our study showed that the phosphorylation of NF-κB, which has been shown to participate in NETs formation (19), is not involved in DXM-modulated NETosis. It may be because different stimuli were used, with bacteria in ours and PMA in others. Nevertheless, as we only detected the phosphorylation of NF-κB in whole cell, it could not be excluded that there were NF-κB shifting from plasma to nucleus.

The specific detection of microorganisms by innate cells is mediated by PRRs—germline-encoded receptors that recognize microbial structures referred to as pathogen-associated molecular patterns (26). TLRs are essential PRRs that mediate the recognition of microbial structures, such as those of bacteria, as well as the subsequent inflammatory and adaptive responses (27–30). Because neutrophils and TLRs are, respectively, the prototypical cells and receptors involved in innate immune responses, the effect of TLRs on NETosis was investigated. Our findings suggested that TLRs involved in inflammatory response could be key regulatory factors in NETs formation. Our results showed that *S. aureus*-induced NETosis was markedly inhibited by TLR2 and TLR4 antagonists and enhanced by TLR2 and TLR4 agonists. This strongly supports the role of TLR2 and TLR4 in the biogenesis of NETs, but these effects were not observed in PMA-induced NETosis. Furthermore, neither TLR5 nor TLR6 agonists/antagonists had any effect on bacteria-induced NETosis. As TLR2 is the main receptor for Gram positive, and TLR4 is for Gram-negative bacteria, respectively, it is reasonable that both of them may directly or indirectly participate in the process of NETosis triggered by *S. aureus* through the whole inflammatory network. It is further confirmed by the following results. The addition of TLR2 and TLR4 agonists (HKLM and LPS) rescued DXM-inhibited NETs formation induced by *S. aureus*, but to a lower extent than in the control group stimulated by *S. aureus*. Therefore, we believe that both TLR2 and TLR4 were involved in DXM-modulated NETosis, which is consistent with the observation in other studies that multiple receptors may together regulate NETs formation (31). Besides, we were unable to conclude whether other TLRs that mediated the interaction of neutrophils and other pathogens like viruses could also be involved.

In addition, we aimed to determine the relationship between DXM and TLRs. Both HKLM and LPS rescued DXM-reduced NETs formation. Moreover, neither TLR2 nor TLR4 antagonist could further decrease DXM induced NETosis reduction. This indicated the involvement of TLRs in DXM-reduced NETosis. A previous study showed that DXM down-regulates TLR4 mRNA expression in neutrophils (32), which implies that it may regulate NETosis by modulating TLR expression (33).

Our study has a limitation: we examined neutrophil function only *in vitro*; further *in vivo* studies are needed to characterize the fate of neutrophils. It is also not clear how DXM and TLRs cooperatively modulate NETs formation. Further research is needed to clarify these points.

In conclusion, we have demonstrated that NETs formation can be induced in neutrophils by different stimuli but not by a common mechanism. The mechanism of how DXM modulates bacteria-induced NETs formation was found to be unrelated to oxidant production and phosphorylation of NF-κB. TLR2 and TLR4 are involved in the formation of NETs. Although the specific mechanisms of how DXM regulates NETs formation are unclear, it is possible that DXM regulates NET formation induced by *S. aureus via* a TLR-dependent mechanism.

## Author Contributions

TW and YZ wrote the main manuscript text. YZ, TW, FF, and RH performed the experiments. YZ and TW prepared Figures 1–6. XJ designed the study and provided advice on the discussion.

## Conflict of Interest Statement

The authors declare that the research was conducted in the absence of any commercial or financial relationships that could be construed as a potential conflict of interest. The reviewer SS and handling Editor declared their shared affiliation, and the handling Editor states that the process nevertheless met the standards of a fair and objective review.
